# Development of an HTS-SQUID-Based Receiver for Long-Range Magnetic Induction Communication in Extreme Environments

**DOI:** 10.3390/s23094434

**Published:** 2023-04-30

**Authors:** Yulong Li, Tiequan Xu, Yue Wang, Furen Wang, Zizhao Gan

**Affiliations:** Applied Superconductivity Center and State Key Laboratory for Mesoscopic Physics, School of Physics, Peking University, Beijing 100871, China

**Keywords:** magnetic induction communication, underwater communication, high temperature superconducting quantum interference devices, flux transformer, communication range, magnetic field sensitivity

## Abstract

The communication range of magnetic-induction (MI) technology in extreme environments such as underwater or underground is limited by the dipole-like attenuation behavior of the magnetic field as well as the eddy current induced loss in conductive media, and therefore a highly sensitive receiver is generally required. In this work, we propose the use of a highly sensitive superconducting quantum interference device (SQUID) in MI communication and try to provide a comprehensive investigation on developing a SQUID-based receiver for practical MI applications. A portable receiver scheme integrating a SQUID sensor and a coil-based flux transformer was proposed. The high sensitivity and long-range communication capability of the proposed receiver was experimentally demonstrated by spectroscopic measurements and reception experiments on a receiver prototype. Based on the experimental demonstrations, the sensitivity optimization of the proposed scheme was further investigated by simulation studies, which suggest that a communication distance exceeding 100 m and a channel capacity of ∼20 kb/s in underwater environment could be achieved based upon the optimization of the developed prototype. The results presented in this work have highlighted the potential of deploying SQUID sensors for long-range MI applications in extreme environments.

## 1. Introduction

Electromagnetic (EM) wave-based terrestrial communication technology is a building block of modern societies. This technology features a high data rate by generally employing carriers with high frequencies to expand the useable communication channel bands and has been commonly adopted in state-of-the-art communication systems. While this technology has exhibited a long transmission range and a high communication rate for terrestrial communication, when deployed in extreme scenarios such as underground or underwater communication, the EM technology faces severe challenges. In such harsh environments, the high-frequency EM waves have violently suffered from attenuation due to the profound absorption by conductive mediums, which makes long-range communication in such environments almost impossible. Moreover, because of the complicated distribution of the mediums (underground rocks and soils, or different ocean flows) as well as their multi-variable-dependent (temperature, pressure, etc.) and time-varying dielectric properties [1], the EM waves have confronted uncontrollable multi-path fading, resulting in signal distortion due to the reflection or dispersion when crossing the interfaces of different mediums. In this case, it has raised uncertainty in space and time for the transmission of EM waves, which makes it difficult to guarantee a reliable communication channel using EM technology.

Magnetic induction (MI) communication technology, which has been proposed and developed in recent years, is now considered as a reliable and low-power-consumption substitute for traditional EM communication in radio frequency (RF) challenging environments, and has attracted great interest from many research communities [2,3,4,5,6,7]. This technology employs a magnetic field as the carrier instead of traditional EM waves for data transmission. The basic principle of MI communication is to utilize the mutual inductance between the transmitter and the receiver (both can be loop coils) to establish communication links, and to modulate the AC currents within the transmitter coil to realize data encoding. There are several advantages for deploying this technology, including negligible multi-path fading, which ensures predictable and reliable channel conditions, and low signal attenuation caused by eddy current loss, which may greatly expand the accessible communication range in lossy media. The above superiorities of this technology beyond the traditional EM technologies lie in the fact that the permeability of most substances in nature remains almost the same as that of the free space [8], which is in contrast with their dielectric constants. However, those advantages of MI technology are lost when the communication distance is larger than the so called non-radiative near-field region, which is defined by $d_{nf}=\lambda_{m}/2\pi$, where $\lambda_{m}$ represents the wavelength of the signal in a medium. When considering long-range MI communication, this boundary is of great importance since the signal propagation characteristic beyond this boundary would be converted into EM behavior and suffer from severe attenuation in RF-challenging environments in the same way as EM waves [6,8]. Therefore, to maximize the communication range, the channel bands for MI technology are typically limited to low frequencies. For example, to achieve a transmission distance of higher than 500 m, it is better to use the frequency band of below 100 kHz according to the near-field restriction [9]. Another important feature that hinders the deployment of MI technology in long-range communication is the dipole-like magnetic field characteristics, whose attenuation follows $1/d^{3}$ behavior with increasing transmission distance, which decays much faster than 1/d of EM waves in the near-field region [6,7]. The above characteristics together have greatly limited the accessible communication range of MI technology. Therefore, a wideband receiver technology featuring high sensitivity at a low frequency band within several tens of kHz is generally required for the implementation of long-range MI communication.

It is noted that some relay technologies have been proposed to extend the communication range based on transmission between traditional loop coils [10,11,12]. Alternatively, another way to address the range limitation problem is to employ more sensitive magnetic field sensors as MI receivers in place of the traditional coil. Very recently, some highly sensitive sensors have been employed in MI communications, including sensors based on anisotropic-magnetoresistance (AMR) [13,14] and giant-magneto-impedance (GMI) effects [15,16,17], as well as optically pumped magnetometers (OPMs) [18]. In addition to the above sensor technologies, superconducting quantum interference devices (SQUIDs), which are well known as a very sensitive detector of magnetic flux and consist of Josephson junctions closed with a superconducting loop [19], could be another very promising candidate for the implementation of long-range MI communication. The advantages of the SQUID over the other sensor technologies as an MI receiver are presented as follows: (1) The ultra-high sensitivity. As a near-quantum limited sensor, SQUID owns the capability for detecting weak signals down to the femto-Tesla ($10^{-15}$ T, fT) level and has demonstrated various applications such as in biomagnetism and nondestructive evaluation, which require very high field sensitivity [20,21,22,23]. The field-detection ability of the SQUID sensor is better than AMR- or GMI-based sensors whose state-of-the-art sensitivity remains at the pico-Tesla ($10^{-12}$ T, pT) level. (2) The appropriate detection bandwidth. As a wide-band receiver, the SQUID typically has a bandwidth of 100 kHz, which is in line with the bandwidth requirements for implementing MI applications. The SQUID bandwidth is much larger than the typical value of 1 kHz [18] of the OPM sensors, which leads to a higher channel capacity. Moreover, SQUID sensors have typically featured low 1/f-like noise (i.e., flicker noise), while this type of noise in a traditional coil-based receiver can be considerably large. The above discussions have highlighted the value of deploying SQUID sensors in MI communication in extreme environments to extend the communication range. Except for the above presented merits, there are also several disadvantages that remain to be addressed when deploying SQUID technology. Here, one of the major concerns lies in the mobility problem. The operation of the SQUID generally requires a very low temperature, where additional cooling setup or equipment is needed. This could greatly increase the size of the whole SQUID receiver system. Moreover, the high sensitivity accompanying a limited dynamic range often requires SQUID to be properly shielded inside a magnetically shielded room (MSR). The above two problems, to a certain extent, have hindered the deployment of the SQUID sensor in practical long-range MI applications.

The main objective of this work is to introduce SQUID technology into the MI research community by addressing the above two problems and to undertake a comprehensive investigation of the SQUID-based MI communication in order to show the great competitiveness of the SQUID among the exiting receiver technologies using highly sensitive sensors. For this purpose, a portable receiver scheme intended for practical long-range MI communication applications employing high-transition-temperature superconducting (HTS) SQUID sensors was proposed and developed in this work.

The remainder of this article is organized as follows. Section 2 describes the basic conception and related theoretical background for deploying SQUID sensors in MI communication. Section 3 details the practical implementation of a testbed communication system integrating the developed SQUID receiver. Experimental demonstrations on the constructed receiver prototype are presented in Section 4. The sensitivity optimization and communication range extension issues of the proposed SQUID receiver are addressed by conducting simulation investigations. This work highlights the great potential of deploying highly sensitive HTS-SQUID sensors in practical long-range MI applications.

## 2. MI Communication Employing HTS SQUID Sensor

In this section, the conceptualization of employing SQUID sensor in MI communication is introduced, and a practical receiver scheme is proposed. The basic principles and theoretical backgrounds related to the proposed receiver scheme are described, and the formulas necessarily needed for theoretical calculations in this work are presented.

### 2.1. Conceptual Scheme Design

As had been discussed in previous section, the sensitivity superiority of the SQUID has made it a very promising sensor technology for implementing long-range MI communication in extreme environments, if the mobility limitations can be properly addressed. For a conventional SQUID sensor made from low-transition temperature (LTS) superconductors whose operation generally requires an extreme temperature of 4 K (i.e., −269 °C) provided by liquid helium (LHe). The holding time of LTS-SQUID operation could be a big problem due to the low heat of evaporation of liquid helium and therefore a large-sized cryostat with thick thermal insulation may be required, which could reduce the system mobility. However, with the advent of high-transition temperature superconductors, e.g., YBa${}_{2}$Cu${}_{3}$O${}_{7-\delta }$ (YBCO), the operation of the SQUID sensors under liquid nitrogen (LN${}_{2}$) temperature of 77 K has been promised. The heat of evaporation of LN${}_{2}$ is a factor of 60 higher than that of LHe, the LN${}_{2}$ is much cheaper and safer to handle, and the cryostat or cryocooler for 77 K can be much more compact and lighter than that for 4 K [19]. All of these features provide the opportunity to significantly lessen the cooling burden and enhance the mobility of the system in the operation of the SQUID with HTS sensors [19,24]. In addition, a high-performance magnetic shield comparable with a traditional MSR is now available with a small-sized superconducting shield tube made from HTS materials. Under this circumstance, we propose a portable receiver scheme combining HTS-SQUID sensor and HTS magnetic field shield. Since the SQUID sensor inside the magnetic shield could not effectively detect the external magnetic fields, a flux transformer is introduced here to transfer external magnetic field signals into the shielded SQUID. With such a configuration, the proposed receiver scheme can effectively detect external signals utilizing the inductive coupling between SQUID and the flux transformer while still featuring the low noise characteristics of the SQUID sensor. Such a receiver scheme could potentially be used as a sensitive receiver in MI communication applications, for instance, in underwater or underground environments for underwater surveillance, inshore communication among stationary or mobile nodes, communication among underground tunnels or mines, and soil condition monitoring. In the following subsections, we further consider the practical implementation of the above proposed receiver scheme using a copper conductor flux transformer.

### 2.2. Receiver Noise Model

**Figure 1:**
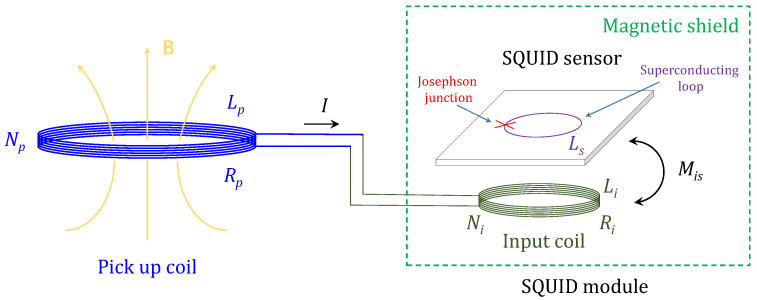
Schematic diagram of the proposed receiver integrating a pick-up coil and a SQUID module. The pick-up coil has inductance $L_{p}$, resistance $R_{p}$, and number of turns $N_{p}$. The SQUID sensor, where the red cross represents the Josephson junction inserted into the superconducting loop with inductance $L_{s}$, is inductively coupled through mutual inductance $M_{is}$ with the input coil, which has inductance $L_{i}$, resistance $R_{i}$, and number of turns $N_{i}$, and both of them are magnetically shielded, constituting the SQUID module.

The noise performance is of particular importance for an MI receiver. When a SQUID and a copper conductor-based flux transformer are coupled to form the receiver, the noise characteristics of the combination can be quite different from that of their individual parts. Here, we introduce a simple noise model similar to that proposed in [25] for evaluating the sensitivity of the proposed SQUID receiver scheme. Supposing that the SQUID had been properly shielded according to the above introduced scheme, the noise of the receiver would mainly arise from two sources: (1) The intrinsic noise of the SQUID; (2) The thermal noise generated from the conductor of flux transformer that couples into the SQUID. Figure 1 depicts a schematic diagram of the key components of the proposed receiver, including a SQUID sensor and a flux transformer with both pick-up coil and input coil made of conventional copper conductors.

The thermal noise voltage $V^{2}=4k_{B}TR$ across the coil resistance *R* at a finite temperature *T* will produce a noise current $I_{N}$ threading the flux transformer. Therefore, a noise flux $M_{is}I_{N}$ is induced within the SQUID loop from the input coil via mutual inductance $M_{is}$. The total flux noise of the SQUID based on the above model, which is equivalent to the noise of the receiver, can then be expressed in a root-sum-square form [19], known as flux noise spectral density (in unit of $\Phi_{0}/\sqrt{\mathrm{Hz}}$, where $\Phi_{0}\approx 2.7\times 10^{-15}$ Wb is the magnetic flux quantum):

(1) $$S_{\Phi }^{1/2}\left(f\right)=\sqrt{{\left(M_{is}\times I_{N}\right)}^{2}+S_{\Phi ,SQ}\left(f\right)}=\sqrt{\frac{4k_{B}TRM_{is}^{2}}{R^{2}+{\left(2\pi fL\right)}^{2}}+S_{\Phi ,SQ}\left(f\right)}$$

where $k_{B}$ represents the Boltzmann constant, and $R=R_{p}+R_{i}$ and $L=L_{p}+L_{i}$, respectively, represent the total resistance and inductance of the flux transformer. The first term on the right-hand side of the equation represents the noise contributed by the transformer resistance, and the second term $S_{\Phi ,SQ}\left(f\right)$ represents the intrinsic flux noise of the SQUID.

Similarly, when an external magnetic field changing with frequency *f* is presented in the pick-up coil, the flux transformer will transfer a time-varying magnetic flux to the SQUID loop [25]. The effective area $A_{eff}$ of the receiver, which describes the efficiency of transforming an external magnetic field into a detectable flux signal to SQUID, is given by (in units of mm${}^{2}$):

(2) $$A_{eff}=\frac{\Delta \Phi }{\Delta B}=\frac{2\pi^{2}r_{p}^{2}N_{p}M_{is}f}{\sqrt{R^{2}+{\left(2\pi fL\right)}^{2}}}$$

where ΔB and ΔΦ, respectively, represent the change of the external magnetic field and the corresponding flux generated in the SQUID ring with respect to this change, while $r_{p}$ and $N_{p}$, respectively, represent the radius and the number of turns of the pick-up coil. One could infer from the equation that the effective area of the coupled receiver for the high-frequency signal is independent of frequency; when the frequency is smaller than the threshold value, which is called the corner frequency, the resistance term becomes important in determining the effective area and the transfer efficiency declines with decreasing frequency.

Based on Equations (1) and (2), the magnetic field noise spectra density of the receiver is expressed as (in unit of T$/\sqrt{\mathrm{Hz}}$):

(3) $$S_{B}^{1/2}\left(f\right)=S_{\Phi }^{1/2}\left(f\right)/A_{eff}=\sqrt{\frac{4k_{B}TR}{N_{p}^{2}\;{\left(\pi r_{p}^{2}\right)}^{2}\;{\left(2\pi f\right)}^{2}}+S_{\Phi ,SQ}\left(f\right)\times \frac{R^{2}+{\left(2\pi fL\right)}^{2}}{N_{p}^{2}\;{\left(\pi r_{p}^{2}\right)}^{2}\;{\left(2\pi f\right)}^{2}M_{is}^{2}}}$$

The $S_{B}^{1/2}\left(f\right)$ determines the minimum magnetic field signal detectable by the receiver if one assumes a signal-to-noise ratio (SNR) of 0 dB, that is, the strength of the magnetic field signal is equal to that of the magnetic field noise. In light of this, in the literature the $S_{B}^{1/2}\left(f\right)$ is often referred to simply as the magnetic field sensitivity of the SQUID [19,26]. Following this convention, in this article we may call $S_{B}^{1/2}\left(f\right)$ the magnetic field noise or magnetic field sensitivity interchangeably. From Equation (3), one can see that the sensitivity of the above-proposed receiver scheme depends on a set of parameters of both the SQUID and the pick-up coil. Given a particular SQUID sensor, to make the most of the high sensitivity of the SQUID, a careful inspection of the flux transformer design is critical for obtaining a highly sensitive MI receiver.

### 2.3. Channel and Communication Range Evaluation

For practical implementation, a precise channel modeling is of great importance for one to gain knowledge about the transmission characteristics of a generated magnetic signal when deploying an MI communication. For point-to-point communication between a transmitter and a SQUID receiver, the signal channel can be modeled using a simple magnetic field analysis method [14].

Conceptually, in a long-range transmission, the transmitter coil can be regarded as a magnetic dipole loop antenna driven by currents. Consider a squared coil transmitter with side length $l_{t}$ and coil turns $N_{t}$; the magnetic field intensity *B* produced by a current *I* with frequency *f* flowing through the coil can be calculated by adding together a contribution generated by four segments of the coil [5,27]. Considering a signal transmission along the normal direction of the coil in a non-conductive environment, the magnetic field intensity *B* at a distance *d* can be derived as:

(4) $$B=\frac{2\mu_{0}IN_{t}l_{t}^{2}}{\left(l_{t}^{2}+d^{2}\right)\sqrt{2l_{t}^{2}+d^{2}}}$$

Note that for a large transmission distance ($d\gg l_{t}$), the magnetic field decays as $1/d^{3}$, which is in consistent with the behavior of a dipole.

When the signal transmission is performed in an extreme environment like sea water, an additional field attenuation is caused by the eddy-current-induced loss [24]. By taking into account such an attenuation, the magnetic field intensity presented in Equation (4) can be extended by adding the eddy-current related term:

(5) $$B_{c}=\frac{2\mu_{0}IN_{t}l_{t}^{2}}{\left(l_{t}^{2}+d^{2}\right)\sqrt{2l_{t}^{2}+d^{2}}}e^{-\frac{\sqrt{d^{2}+l_{t}^{2}}}{\delta }}$$

Here, δ represents skin depth of the medium, which depends on the medium properties and transmitter signal frequency *f*. To specify the attenuation of magnetic field transmitted from the transmitter coil to the SQUID receiver, the magnetic field path loss (MFPL) as a function of *d* is introduced [14] and derived from Equation (5) as:

(6) $$MFPL\left(d\right)=20\log_{10}\frac{B_{c}\left(0\right)}{B_{c}\left(d\right)}=20\log_{10}\frac{\left(l_{t}^{2}+d^{2}\right)\sqrt{2l_{t}^{2}+d^{2}}e^{-\frac{l_{t}}{\delta }}}{\sqrt{2}l_{t}^{3}e^{-\frac{\sqrt{l_{t}^{2}+d^{2}}}{\delta }}}$$

Considering the transmission of the single frequency channel with a bandwidth of 1 Hz, the maximum communication distance $d_{c}$ between the transmitter coil and the SQUID receiver may be determined by equating the level of the magnetic field signal at $d_{c}$ with that of the magnetic field noise, namely, with an SNR of 0 dB. With the driving current $I_{0}$ at frequency $f_{0}$, the magnetic field at $d_{c}$ is then given by:

(7) $$B_{c}\left(I_{0},d_{c}\right)=S_{B}^{1/2}\left(f_{0}\right)\times \sqrt{1Hz}$$

If one utilizes a receiver bandwidth of Δf, then the channel capacity or communication rate (in unit of b/s) can be estimated by the following expression based on the well-known Shannon-Hartley theorem [28]:

(8) $$C=\Delta f\times \log_{2}(1+\frac{B_{c}^{2}}{S_{B}\Delta f})$$

## 3. System Implementation

**Figure 2:**
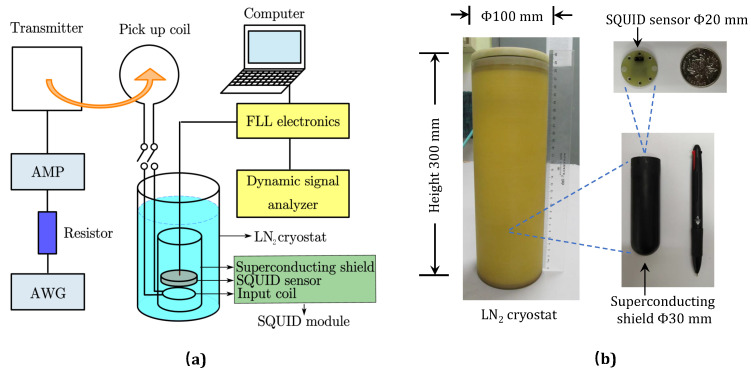
The implemented MI communication prototype system using HTS SQUID as the receiver. (**a**) Schematic diagram illustrating the main components of the system. (**b**) Images of the LN${}_{2}$ cryostat and the key components of the SQUID module: the SQUID sensor and the HTS magnetic shield tube. To better perceive their dimensions, the LN${}_{2}$ cryostat is compared with a ruler (300 mm in length), the SQUID sensor is compared with a coin (Φ24 mm), and the HTS shield is with a ballpoint pen (about 140 mm in length).

To verify the proposed scheme, an MI communication system, including a SQUID receiver, a transmitter, and a laboratory apparatus, was constructed as depicted in Figure 2. A SQUID receiver prototype (a SQUID coupling with a flux transformer according to the above proposed scheme) was implemented. In the following, the fabrication and construction; their key characteristics; and the arrangements of the main components of the system, including the HTS SQUID sensor, the flux transformer, and the transmitter, are detailed respectively.

### 3.1. HTS SQUID Module

The SQUID receiver prototype consists of a pick-up coil, a liquid nitrogen cryostat, and a SQUID module which includes a SQUID sensor, an input coil, and a superconducting magnetic shield tube, as shown in Figure 2. An HTS SQUID sensor fabricated from YBa${}_{2}$Cu${}_{3}$O${}_{7-\delta }$ (YBCO) following the steps detailed in previous reports [29,30,31,32] is applied throughout this article. The SQUID sensor has a package size of Φ20 mm × 4 mm and a frequency-independent effective area of 0.67 mm${}^{2}$ [29]. An input coil, which shall be detailed in Section 3.2, is closely stacked to the SQUID sensor to achieve the best possible coupling, and both of them are mounted inside a small HTS magnetic shield tube (Φ30 mm × 100 mm), where they all together form the SQUID module. A magnetic shielding factor of higher than $10^{6}$ of the HTS tube could sufficiently prevent the SQUID sensitivity from deteriorating when placed in an environment with large external magnetic interferences. The SQUID module is operated at a temperature of 77 K inside an epoxy LN${}_{2}$ cryostat (Φ100 mm × 300 mm) and is connected to the room temperature flux-locked loop (FFL) electronics (in box with size of about 170 mm × 120 mm × 80 mm) for signal readout. Details on the FFL scheme for the SQUID signal readout may be found in [19,26]. In brief, with the FLL, the magnetic flux change in the SQUID induced by the magnetic field noise or signal is exactly compensated via a feedback circuit, and, consequently, the SQUID is maintained at a fixed working point, that is, it is “flux-locked”. The voltage output of the FLL (specifically the feedback circuit) then corresponds to the magnetic flux change in the SQUID, and, with the parameters of the feedback circuit, the flux-to-voltage transfer coefficient ∂V/∂Φ can also be specified; these, together, enable us to determine the magnetic flux noise or signal measured by the SQUID. In other words, the SQUID flux noise $S_{\Phi }=S_{V}/{\left(\partial V/\partial \Phi \right)}^{2}$, where $S_{V}$ represents the voltage noise at the output of the FLL. This holds regardless of whether or not there is a flux transformer integrated with the SQUID. From this, one may also notice that the determined $S_{\Phi }$ has actually included the noise contribution from the FLL electronics as the $S_{V}$ is measured at the output of the FLL, although it has been shown that the FLL noise contribution to the total flux noise is usually negligible [19]. In this article, when we refer to $S_{\Phi ,SQ}\left(f\right)$, namely, the intrinsic flux noise of the SQUID, in principle it also involves the FLL noise contribution because it was experimentally measured but not calculated, and we just used this measured value as an input in Equation (1) or (3) for further calculation. In the present work, the flux-to-voltage transfer coefficient ∂V/∂Φ is specified as 170 mV/$\Phi_{0}$, and a dynamic signal analyzer has been used to capture and record the FLL readout signal from the SQUID. Finally, the magnetic field noise $S_{B}$ is converted from $S_{\Phi }$ through $S_{B}=S_{\Phi }/A_{eff}^{2}$ with the effective area $A_{eff}=\Delta \Phi /\Delta B$. For the SQUID without integrating the flux transformer, the $A_{eff}$ is measured by monitoring the magnetic field change ΔB with a magnetic field calibration coil when the flux of the SQUID increases a flux quantum $\Delta \Phi =\Phi_{0}$ [29]. When the SQUID integrates with the flux transformer, the $A_{eff}$ is calculated from Equation (2) and confirmed by the signal reception measurements by referencing the signal strength from the transmitter, as described in detail later.

### 3.2. Flux Transformer

When the SQUID is coupled with an external flux transformer made from conventional copper conductor, the frequency-independent effective area of the SQUID receiver will be modified, which depends on the transformer geometry. To make the field sensitivity of the proposed receiver as high as possible, according to Equation (3), the mutual inductance $M_{is}$ should be designed to be as large as possible while keeping the relatively small resistance of the flux transformer. For this purpose, a flux transformer with coil geometry parameters of $r_{p}=25$ mm, $N_{p}=10$, $r_{i}=10$ mm, and $N_{i}=100$ was fabricated from copper wires with two different diameters. The key parameters of the fabricated flux transformer are specified in Table 1. The resistance of the input coil is measured to be 17.4 Ω at a room temperature of 300 K, and when immersed in liquid nitrogen to couple with the SQUID sensor, the corresponding resistance at 77 K is decreased to 2.6 Ω. The pickup coil is extended out from the liquid nitrogen cryostat to a room temperature environment, and the resistance is measured to be 0.1 Ω.

The mutual inductance between the input coil and the SQUID can be estimated by using the following formula from [2]:

(9) $$M_{is}=\frac{\mu_{0}\pi N_{i}N_{SQ}r_{i}^{2}r_{SQ}^{2}}{2\sqrt{{\left(r_{i}^{2}+d_{is}^{2}\right)}^{3}}}$$

Here, $\mu_{0}$ represents permeability of free space, $N_{SQ}$ represents the coil turns of the SQUID which equals to 1, and $d_{is}$ is the distance between input coil and SQUID. We have estimated the mutual inductance by putting the SQUID effective area of 0.67 mm${}^{2}$, the input coil geometry parameters, and the average distance between the SQUID and the input coil into Equation (9), and we obtained a value of 3 nH.

It is worth noticing that the pick-up coil in our design can be disconnected from the SQUID module via an SMA connector, which ensures the flexibility of implementing coils with different geometries for further receiver optimization.

### 3.3. Transmitter System

A squared coil transmitter with loops $N_{t}$ of 3 and side length $l_{t}$ of 500 mm was built by winding a multi-conductor cable onto a wooden frame. The main characteristics are also listed in Table 1. The inductance of the squared coil is estimated by using the formula presented in [33]. Since the resistance of the transmitter in this case is very small (i.e., smaller than 0.1 Ω), to ensure the transmitter a wide-band emission, an additional 5 Ω resistor was connected in series with the transmitter. With such transmitter parameters, a flat response with respect to different driving frequencies within our SQUID-detection bandwidth (∼20 kHz) can be guaranteed down to very low frequencies, which is suitable to use for MI communication. An arbitrary waveform generator (AWG) is connected to the transmitter as signal source for transmission tests. The signal currents flowing through the series resistor are then amplified by a power amplifier (AMP) and drive the transmitter coil.

## 4. Results and Discussion

### 4.1. Experimental Verification of Receiver Prototype

#### 4.1.1. Noise Characterization

To evaluate the receiver performance, firstly, the noise characteristics of both the SQUID sensor and the receiver prototype presented above have been investigated by means of spectroscopy measurement. The measurements were conducted at daytime in a laboratory located right next door to a subway station in an urban area. The noise spectra were measured by a dynamic signal analyzer and recorded in the frequency domain for further analysis.

**Figure 3:**
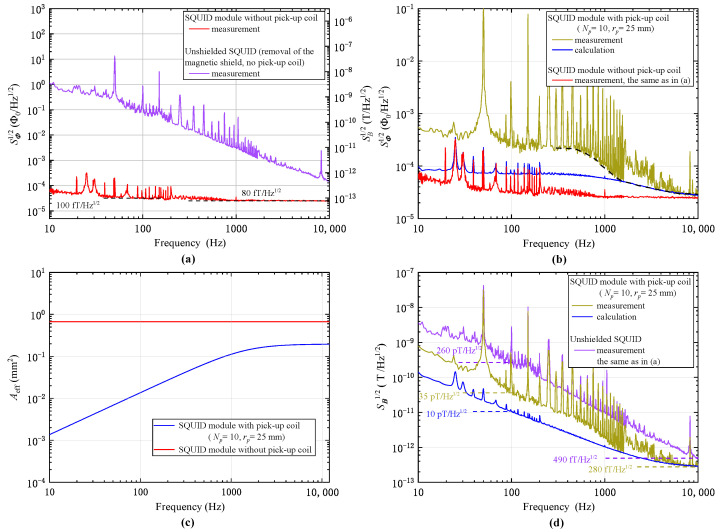
Spectroscopic characterization of the implemented receiver prototype (SQUID module). (**a**) The noise spectrum of the prototype before pick-up coil connection. For comparison, the noise spectrum of the unshielded SQUID with removal of the magnetic shield is also plotted. (**b**) Comparison on flux noise spectra of the prototype before and after the connection of pick-up coil. (**c**) The effective area $A_{eff}$ of the prototype as a function of frequency calculated from Equation (2) compared with that of the SQUID measured without connection of the pick-up coil. (**d**) Comparison of magnetic field noise of the receiver prototype and the unshielded SQUID.

As discussed above and shown by Equation (1), both the intrinsic noise of the SQUID sensor, $S_{\Phi ,SQ}\left(f\right)$, and the noise of the copper flux transformer contribute to the noise of the receiver prototype. Therefore, to calculate the noise of the receiver prototype from Equation (1) or (3) to conduct a comparison with the measurements, the $S_{\Phi ,SQ}\left(f\right)$ needs to be determined in experiments, which can also indicate the quality of the fabricated SQUID sensor. To evaluate $S_{\Phi ,SQ}\left(f\right)$, the noise spectrum of the SQUID module before connecting with the pick-up coil was recorded, as depicted in Figure 3a. In this case, the SQUID sensor is under magnetic shielding as it is placed inside the HTS magnetic shield tube; hence, the SQUID is essentially decoupled from external environmental noise and the recorded spectrum can be regarded as representing the intrinsic noise of the SQUID. This is analogous to other $S_{\Phi ,SQ}\left(f\right)$ measurements that place the SQUID sensor in an MSR [19]. As can be seen in Figure 3a with the red line, the SQUID showed a white noise characteristic without the presence of 1/*f* noise within the frequency band of 100Hz–10kHz, where $S_{\Phi ,SQ}^{1/2}\left(f\right)$ of 27 $\mu \Phi_{0}/\sqrt{\mathrm{Hz}}$ at 10 kHz and 33 $\mu \Phi_{0}/\sqrt{\mathrm{Hz}}$ at 100 Hz are shown, which correspond to magnetic field sensitivity of 80 fT/$\sqrt{\mathrm{Hz}}$ at 10 kHz and 100 fT/$\sqrt{\mathrm{Hz}}$ at 100 Hz, respectively. We note that this field sensitivity in the white noise region is comparable to that (50–130 fT/$\sqrt{\mathrm{Hz}}$) reported recently for HTS SQUIDs in an MSR employed in magnetoencephalography (MEG) measurements [21], suggesting the good quality of our fabricated SQUID sensor and its potential in applications, for example, MEG, for the direct detection of weak magnetic fields in an MSR without flux transformers.

Figure 3a also shows the noise spectrum (violet line) of the SQUID sensor with the removal of the HTS magnetic shield tube, that is, under no magnetic shielding and accordingly denoted as the unshielded SQUID. In this case, the SQUID sensor was directly exposed to the external environment with rather strong electromagnetic interferences, and it is seen that the noise level of the SQUID increased significantly compared with the intrinsic one in red, exhibiting a 1/*f*-like dependence on frequency. At 10 kHz, the field noise witnessed a considerable increase from the intrinsic value of 80 fT/$\sqrt{\mathrm{Hz}}$ to 490 fT/$\sqrt{\mathrm{Hz}}$. A more dramatic increase in noise from 100 fT/$\sqrt{\mathrm{Hz}}$ to 260 pT$\sqrt{\mathrm{Hz}}$ was found at 100 Hz, which increased by more than three orders of magnitude. In the frequency range from about 100 to 1600 Hz, there were many spikes in the spectrum at frequencies of higher-order harmonics of 50 Hz. We suspect these characteristic peaks originated from the large electromagnetic disturbances caused by nearby instruments or facilities powered with the 50 Hz power line, although the specific origin was difficult to identify because, as aforementioned, the measurements were taken at daytime in a physics building with lots of equipment running simultaneously and with a subway station right next to it [34]. It was also observed in the measurement that sometimes the environmental disturbances can become so large that the unshielded SQUID will become temporally inoperable. This highlighted the need for developing the receiver prototype by magnetically shielding the SQUID and integrating it with the flux transformer.

Figure 3b shows in dark yellow the noise spectrum of the SQUID module with the integration of the flux transformer, that is, with the connection of the pick-up coil. Compared with the spectrum without the pick-up coil, it was observed that the noise became higher and showed a progressive increase with the decrease in frequency and the presence of additional spikes between 200 and 1600 Hz, illustrating the effect of environmental interferences coupled through the pick-up coil. The black dashed line is used to guide the eye to the baseline value of the measured spectrum. At frequencies above 1600 Hz, the deviation of the flux noise from the baseline level could be due to the resolution limitation of the measurement as the fine structure of the spikes presented in this frequency region may have not been well captured. The theoretical calculation from Equation (1) using the SQUID intrinsic flux noise presented in Figure 3a and the coil parameters specified in Table 1 is also shown with a blue line for comparison. As can be seen, at a frequency higher than about 3 kHz, the measured spectrum fits well with the calculation. The agreement between experiment and theory indicates the accuracy of our calculation and validates the effectiveness of the previously presented model in describing the noise characteristics of the implemented receiver prototype. For a frequency lower than about 1.6 kHz, the measurement and the calculation show an apparent discrepancy with the calculated noise that is considerably lower than the measured one. We believe this should be attributed to the presence of excess noise in the measurement arising from the environmental electromagnetic interferences that are not included in the calculation. As mentioned above, the strong environmental interferences could be coupled with the SQUID via the pick-up coil, and such excess noise often features broadband behavior at low frequencies [34].

The effective area of the SQUID module after the connection of the pick-up coil calculated from Equation (2) is shown in Figure 3c as a function of frequency. As shown in the figure, the effective area of the receiver prototype is smaller than that of the SQUID sensor measured with no pick-up coil. In the high-frequency region, the effective area shows frequency-independent behavior, while in the low-frequency region starting from about 3 kHz, the effective area falls off rapidly with the decrease in frequency. The effective area of the receiver prototype shows a value of about 0.19 mm${}^{2}$ or 0.013 mm${}^{2}$ at 10 kHz or 100 Hz, respectively, which is about 3 or 50 times smaller than that of the single SQUID. This indicates a reduced magnetic field-to-flux transfer coefficient of the receiver compared with the single SQUID when detecting low-frequency signals. Here, we emphasize the veracity of the calculated effective area, which will be verified in Section 4.2.

The magnetic field noise of the receiver prototype was evaluated using Equation (3) and compared with that of the unshielded SQUID, as displayed in Figure 3d. It is shown that the field noise of the receiver prototype is notably reduced from that of the unshielded SQUID. At 10 kHz and 100 Hz, while the unshielded SQUID showed field noise of about 490 fT/$\sqrt{\mathrm{Hz}}$ and 260 pT/$\sqrt{\mathrm{Hz}}$, respectively, the corresponding values for the receiver prototype are 280 fT/$\sqrt{\mathrm{Hz}}$ and 35 pT/$\sqrt{\mathrm{Hz}}$, respectively (280 fT/$\sqrt{\mathrm{Hz}}$ and 10 pT/$\sqrt{\mathrm{Hz}}$, respectively, according to the calculation). This comparison clearly indicates that the receiver prototype would outperform the unshielded SQUID in complex electromagnetic environments due to its better noise property; therefore, it is preferable to be used in practical applications as it warrants a larger receiving distance from the same signal source. Compared with some other sensor technologies such as AMR or GMI that have already been employed in MI communications, the SQUID-based MI receiver has shown great sensitivity advantage, even with the unoptimized receiver prototype.

#### 4.1.2. Reception Experiments

To verify the superiority of the above developed receiver prototype in conducting practical long-range communication in extreme environments, single-channel transmission tests were performed on the communication system implemented in Section 3. Two receiver configurations that had been characterized in Section 4.1.1, i.e., the unshielded SQUID sensor and the prototype, were respectively inspected and compared. During the experiment, signals with a very low frequency of 165 Hz were generated by the transmitter coil driven with different current intensities. The receiver system was placed at a distance away from the transmitter for signal reception.

**Figure 4:**
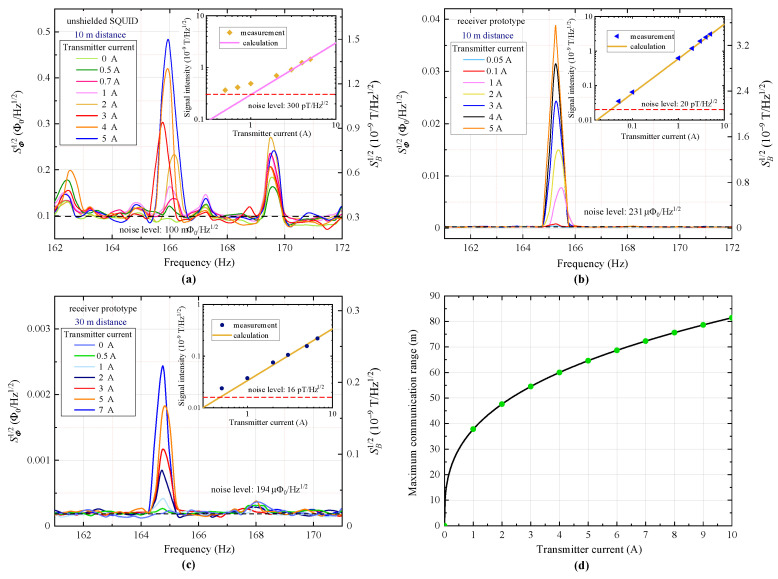
Results from reception experiments. (**a**) Off-axis transmission between the unshielded SQUID sensor and the transmitter coil with a distance of 10 m. (**b**) Co-axis transmission between the receiver prototype and the transmitter coil with a distance of 10 m. (**c**) Co-axis transmission between the receiver prototype and the transmitter coil with a distance of 30 m. Upper right insets present the signal intensity at receiver side from theoretical evaluation and experimental detection. (**d**) Maximum communication range of the constructed system (for SNR at 0 dB) with respect to different transmitter driving currents.

Firstly, the veracity of the theoretical formula for a magnetic field generated by transmitter coil was checked by using the already calibrated SQUID sensor for signal reception. With the HTS magnetic shield removed from the receiver, the magnetic field signal was detected directly by the SQUID sensor via its superconducting loop. Since the SQUID detection has been limited to the direction that is perpendicular to the ground due to the installation restrictions, in this experiment the transmission between the transmitter coil and the pick-up coil of the receiver prototype was conducted in an off-axis way with a 90° misalignment between the two coils at a distance of 10 m. For such a transmission, the field intensity at the receiver side would be about half the case of co-axis transmission according to theoretical evaluation. Figure 4a presents the measured spectra of the reception tests with respect to the different transmitter driving current of I=0–5 A, with the corresponding magnetic signal intensity depicted in the upper right inset. The calculated signal amplitude in receiver side was shown to correspond well with the measurement when a large current was applied, while for a small current the generated signal was buried in a rather high background noise near 165 Hz (≈300 pT/$\sqrt{\mathrm{Hz}}$) due to the degradation of the SQUID in presence of environmental electromagnetic interferences.

Figure 4b depicts the results of a 10 m transmission experiment on the receiver prototype. To achieve the longest possible communication distance, the experiment was conducted in a co-axis way. From the shown results we find that, while the magnitude of the flux signal detected by the prototype is about 25 times smaller than that by the unshielded SQUID with respect to the same source magnetic field, the prototype shows a considerably smaller noise level of 230 $\mu \Phi_{0}$/$\sqrt{\mathrm{Hz}}$, which is about three orders of magnitude smaller than the SQUID. Therefore, the receiver prototype can show much improved detection SNR compared with the direct detection of the SQUID. As a rough estimation, an SNR enhancement of about 25 dB can be achieved if the above two experiments are all conducted in a co-axis way. By fitting the measured data with the calculated signal intensity as shown in the upper-right inset, an effective area of 0.02 mm${}^{2}$ at 165 Hz was obtained for the prototype, very close to the theoretical value of 0.022 mm${}^{2}$ presented in Figure 3c. This demonstrates the accuracy of the theoretical evaluation. To verify the largest possible communication range of our constructed system based on the prototype, a transmission experiment has been further conducted with a larger distance of 30 m. As shown in Figure 4c, the prototype has successfully received the magnetic signals at a minimum transmitter current of about 0.5 A, which corresponds to the transmitter power of ≈1 W. This indicates the long-range communication capability of our developed receiver prototype in extreme environments utilizing low-frequency channels. As an estimation, a channel capacity of several kb/s according to Equation (8) could be expected on our prototype system, if we utilize the full SQUID bandwidth of 20 kHz and a transmitter driving current of 10 A for a 30 m range communication. Figure 4d shows the maximum communication range of the implemented system as a function of the transmitter current, which was estimated based on the above experimental results. As can be seen, an even larger communication range of 81 m can be achieved, but at the cost of a high level of transmitter power of about 500 W (I=10 A). If we further increase the transmitter power, the corresponding range extension could be very limited, as shown by the figure. This has shown the limitation of using a high level of transmitter power to increase the communication range.

The above reception experiments on the receiver prototype have demonstrated the great superiority of employing the proposed receiver scheme for the practical implementation of MI communication. Nevertheless, it has also suggested a limited communication distance with the present prototype design, which could make it hard to achieve an even larger communication range of up to several hundred meters using the constructed communication system at low frequencies. Since the present prototype has a preliminary coil design that might be far from what is optimal, further optimization in terms of sensitivity is needed to boost the maximum communication range.

### 4.2. Simulation Investigations for Receiver Optimization

#### 4.2.1. Sensitivity Optimization

To explore the full potential of the receiver scheme proposed in this work, we have addressed the sensitivity optimization issue in this section by means of simulation based on Equation (3) to show how the pick-up coil parameters influence the receiver noise and to evaluate the best possible sensitivity that could be attained for this receiver scheme. We begin with the calculation of field noise spectra. During the calculation, the geometry parameters of the prototype input coil were used; the variation of conductor resistance for pick-up coil with respect to different geometry parameters at a room temperature of 300 K was considered; and we ignored the ac-resistance effect, which should be negligibly small within the SQUID-detection bandwidth. The inductance of the pick-up coil for calculation was evaluated using the following formula from [27,33]:

(10) $$L=\frac{1}{2}\mu_{0}\pi r_{p}N_{p}^{2}\times G$$

Here, *G* represents the geometry factor, which depends on the coil shapes. During the calculation, we assured a multi-layered short coil geometry with G≈1.

**Figure 5:**
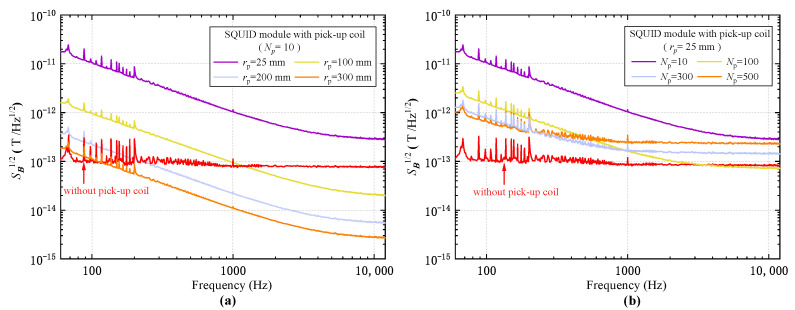
Evolution of magnetic field noise spectra with respect to the variation of (**a**) pick-up coil turns $N_{p}$. (**b**) pick-up coil radius $r_{p}$. The pick-up coil was placed at a room temperature of 300 K. The noise spectrum of the SQUID module without connecting pick-up coils (red line) is shown for comparison.

Figure 5a presents the magnetic field spectra of the receiver with respect to four pick-up geometries with different coil radii $r_{p}$ of 25 mm, 100 mm, 200 mm, and 300 mm, each with the same pick-up coil turns $N_{p}$ of 10. It is shown that, starting from the prototype parameters ($N_{p}=10,r_{p}=25$ mm), with the increase in pick-up coil size, the noise of the receiver decreases progressively, and the sensitivity is considerably improved since the pick-up coil radius has a profound impact on the effective area, as can be inferred from Equation (2). For a portable coil geometry with a radius of 100 mm, a noise level of 21 fT/$\sqrt{\mathrm{Hz}}$ at 10 kHz can be achieved, which is much lower than that of 85 fT/$\sqrt{\mathrm{Hz}}$ of the SQUID sensor. At a low frequency of 100 Hz, a value of 1 pT/$\sqrt{\mathrm{Hz}}$ can be found. Although still higher than that of the SQUID, it is much improved compared with the prototype. As the variation of the coil radius has the same effect on inductance and resistance, the increase in the coil radius has no influence on the corner frequency. Therefore, as is shown in Figure 5a, the receiver noise is reduced in the same manner for each frequency with the increase in pick-up coil radius. It is noted that when the pick-up coil radius is increased to $r_{p}=300$ mm, the field sensitivity of the receiver at 100 Hz begins to surpass the SQUID sensor due to the enhancement of the effective area brought about by the large coil size. Figure 5b shows the spectra of the receiver with respect to four different pick-up coil turns of $N_{p}=$ 10, 100, 300, and 500, with the pick-up coil radius fixed at 25 mm. We notice that, unlike the monotonous decrease with increasing radius, the decrease in noise with increasing coil turns has shown different behaviors at different frequencies. While the low-frequency noise at 100 Hz still decreases monotonously, at a high frequency of 10 kHz the evolution of noise with respect to the coil turns shows a decrease at first from $N_{p}=10$ to $N_{p}=100$ and then a progressive increase from $N_{p}=100$ to $N_{p}=500$. The corner frequency also evolves in the same manner as shown in Figure 5b. Such behavior should be attributed to the coil inductance, as the increase in coil turns has a more profound effect on the inductance than that of the increase in the coil radius.

**Figure 6:**
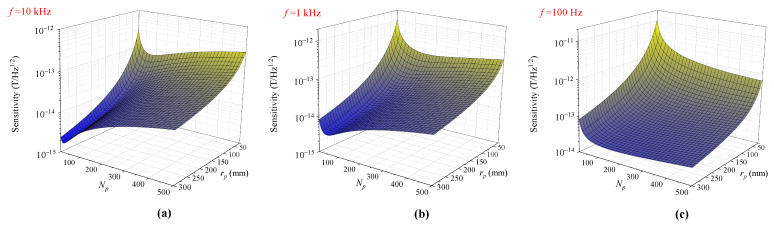
Magnetic field sensitivity as a function of pick-up coil turns $N_{p}$ and pick-up coil radius $r_{p}$ at (**a**) 10 kHz, (**b**) 1 kHz, and (**c**) 100 Hz.

To inspect the evolution of field sensitivity in a more comprehensive way, the field sensitivity $S_{B}^{1/2}$ at frequencies of 10 kHz, 1 kHz, and 100 Hz are plotted as a function of $N_{p}$ and $r_{p}$ and are, respectively, shown in Figure 6. We noticed that, for all of the three frequencies, the sensitivity showed a considerable increase when $r_{p}$ increased, as had been shown in Figure 5a. In contrast with the case of coil radius, the field sensitivity evolves with pick-up coil turns $N_{p}$ in a quite different manner. For a frequency of 10 kHz, it can be noted from Figure 6a that for each given coil radius, by varying the pick-up coil turns, an optimal sensitivity value occurs at a particular value of $N_{p}$ as a “valley bottom”. Exceeding this optimum value, the sensitivity degrades rapidly with the increase in coil turns. When the pick-up coil radius becomes larger, the optimal value of the coil turns becomes smaller. This informs us that, for a large pick-up coil, to achieve the best possible sensitivity at high frequency, the coil turns should not be designed to be too large. In addition, for a large coil radius, a small deviation from the optimal value can result in a profound increase in noise. This indicates a narrowed optimum parameter range for coil turns when designing a large pick-up coil. At a frequency of 1 kHz, while the sensitivity evolution with respect to the coil turns still features non-monotonic behavior, the range restriction is relaxed and the slope of the valley-like optimum region becomes smaller. For a lower frequency of 100 Hz, a much-reduced sensitivity compared with the case of 10 kHz is attained, with the above non-monotonic feature becoming almost invisible. Moreover, when the pick-up coil size becomes larger, the improvement of sensitivity with increasing coil turns becomes slower due to larger coil resistance. For instance, with $r_{p}=300$ mm, the sensitivity variation between $N_{p}=200$ and $N_{p}=500$ is within 20 percent. Therefore, in the practice, for the detection of low-frequency signals, a modest value of coil turns, e.g., 300, is enough to optimize the pick-up coils with different sizes. The optimal coil turns and the best sensitivities accordingly for three different coil sizes ($r_{p}=$ 25, 100, and 300 mm) evaluated from the theoretical calculation are specified in Table 2.

One should notice that the above results are based on a specific SQUID sensor with a particular input coil. In addition to the above pick-up coil optimization, there is still room for sensitivity improvement. For example, by using a SQUID sensor with a lower noise level, the sensitivity of the receiver is expected to be further increased according to Equation (3). If one inspects the equation closely by expressing the field sensitivity as a function of coil turns, it can be found that the above non-monotonous behavior of sensitivity with respect to the coil turns is a result of the competition between coil-related terms and SQUID-related terms. By reducing the SQUID noise level, the adverse increase in the receiver noise at high frequency with large coil turns can be effectively suppressed, which leads to better sensitivity.

#### 4.2.2. Communication Range Evaluation

To verify the possibility of achieving a much larger communication range employing the developed SQUID-based receiver, the extension of maximum communication distance with respect to the optimization of pick-up coil designs was investigated. Here, we have considered two different application scenarios, i.e., terrestrial communication and underwater communication (in sea water).

**Figure 7:**
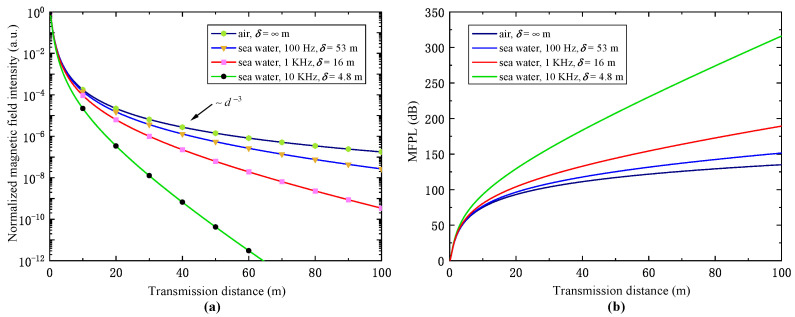
Transmission characteristics of signals generated by transmitter system with different channel frequencies (100 Hz, 1 kHz, and 10 kHz) in the air and in the sea water, calculated based on Equations (5) and (6) assuming the media are homogeneous. (**a**) Normalized magnetic field intensity as a function of transmission distance. Black arrow indicates the 1/$d^{3}$ attenuation behavior of magnetic field in free space. (**b**) Magnetic field path loss as a function of transmission distance.

Prior to range estimation, we have first inspected the transmission characteristics of magnetic signals generated by the transmitter system using Equations (5) and (6). Figure 7 specifies the calculated transmission characteristics for magnetic signals in free space and seawater. The magnetic field intensity and magnetic field path loss (MFPL) as a function of transmission distance are, respectively, shown in Figure 7a,b. The specified value of the skin depth δ used for the calculation for three different signal frequencies (100 Hz, 1 kHz, and 10 kHz) is quoted from [9], which is 53 m, 16 m, and 4.8 m, respectively. We can infer from the results that the attenuation of the magnetic field with respect to the increase in transmission distance generally follows 1/$d^{3}$ behavior in the free space. When the signal is transmitted in sea water, for a transmission distance of 50 m and a channel frequency of 10 kHz, a large magnetic field path loss of 200 dB is shown, which is almost two times that of the case in free space. By decreasing the frequency, the magnetic field path loss has shown a considerable reduction. For the 100 Hz channel, the eddy current-related loss is negligibly small within the near-field region compared with the dipole-behavior-related loss.

**Figure 8:**
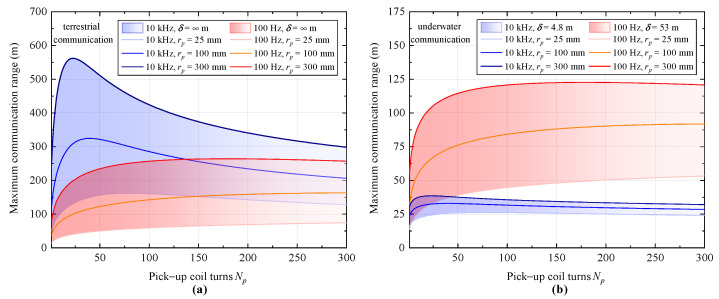
Calculated maximum communication range of the receiver with respect to different pick-up coil radius and turns for (**a**) terrestrial communication and (**b**) underwater communication. As in Figure 7, the media are assumed to be homogeneous in the calculation. The current driving for transmitter coil is set to be 0.5 A. The blue and red areas are used to guide the eye for channel frequencies of 10 kHz and 100 Hz, respectively.

Based on the evaluated channel characteristics, the maximum communication range for terrestrial communication and underwater communication was determined by solving Equation (7) and is, respectively, depicted in Figure 8a,b. During the calculation, the driving current of the transmitter coil is set to 0.5 A. Each of the points within the blue and red areas represents the calculated maximum communication range for channel frequencies of 10 kHz and 100 Hz, within the pick-up coil parameters ranges of $r_{p}=25$–300 mm and $N_{p}=10$–500.

For the case of terrestrial communication as shown in Figure 8a, we have found that without the additional path loss caused by the eddy current, a communication range of several hundred meters could be achieved using the proposed SQUID receiver. Compared with the case of a low channel frequency of 100 Hz, a lager communication range can be achieved with a higher frequency of 10 kHz. As can be seen from the figure, with a large pick-up coil size, $r_{p}=300$ mm, for instance, the receiver could attain a maximum communication range of 560 m, which approaches the near-field boundary of MI communication for 10 kHz channel and is much higher than 260 m at 100 Hz. We have also noticed a much narrower optimum parameter range of $N_{p}$ at a large coil size. Such sensitive behavior of the maximum communication range with respect to coil design is in line with the sensitivity results presented in Section 4.2.1. The theoretical evaluated range for the prototype at a channel frequency of 165 Hz is 37 m, which agrees well with the experimental results from reception experiments presented in Section 4.2.2, when taking into account the influence from the environmental noise. We inferred from the above results that for the terrestrial communication application employing the SQUID receiver, a larger communication range or a higher data rate could be achieved using the high-frequency channels, based upon the careful design of pick-up coils.

For the case of underwater communication, a large eddy current loss presented in sea water can greatly reduce the magnetic field signals transmitted to the receiver, as was shown in Figure 7. As had been expected, the communication range for channel frequency of 10 kHz in sea water has suffered from a profound reduction, as shown by the blue area of Figure 8b. The maximum communication distance for a coil size of $r_{p}=300$ mm has decreased drastically from 560 m to 38 m. Even with a large transmitter power of 500 W (I=10 A), the maximum communication distance is only 49 m. This has generally suggested an upper limit employing our SQUID-based receiver for MI communications in an underwater environment utilizing a carrier frequency higher than 10 kHz. For a lower frequency channel of 100 Hz, a relatively large communication range can be achieved due to the mild eddy current loss for low-frequency signals. By using a large pick-up coil with $r_{p}=300$ mm, according to our calculations, a maximum communication range of about 122 m could be attained, which is about four times larger than that for a carrier frequency of 10 kHz. If a portable pick-up coil size of $r_{p}=100$ mm is used, a maximum communication range of about 92 m could also be reached with a transmitter driving current setting at 0.5 A. As a comparison, the maximum communication range of GMI technology was predicted in [17] to be about 10 m and 3 m in air and seawater, respectively, based on a similar transmitter configuration. The communication range of the traditional coil technology for terrestrial communication was evaluated in [13], and it was shown to be several meters when using a similar-sized transmitter coil and air-core receiver coil. The above results have shown the great advantage of SQUID-based receiver technology at achieving a much-extended communication range compared with other sensor technologies. The channel capacity that could potentially be achieved in an underwater environment for the SQUID-based receiver is evaluated as follows. Under the optimal coil parameters for the pick-up coil with $r_{p}=100$ mm, the noise level utilizing the full SQUID bandwidth of 20 kHz can be estimated to be ≈1 pT. If we fix the communication distance at 100 m, using a transmitter driving current of 10 A and considering the eddy current loss in seawater, the magnetic field intensity at the receiver side calculated from Equation (5) is ≈ 1 pT. Therefore, a data rate of ∼20 kb/s can be achieved in this case according to Equation (8), which is capable of conducting long-range underwater communications with great practical value. The results presented above again highlight the great potential of the SQUID sensors serving as a sensitive MI receiver for long-range applications in extreme environments.

## 5. Conclusions

In summary, in the present work a new approach for MI communication employing a SQUID sensor is introduced, where the ultra-high-sensitive nature of the SQUID is utilized. Comprehensive investigations have been performed on developing a highly sensitive receiver employing SQUID sensors for implementing long-range MI applications. A portable receiver scheme based on a SQUID-flux transformer integrated configuration has been proposed and experimentally implemented. A simple noise model has also been introduced for describing the noise performance of the integrated receiver configuration. The spectroscopy measurement and transmission experiments on the implemented receiver prototype have validated the introduced noise model and demonstrated the long-range communication capability of the proposed receiver for MI applications. Simulation studies have been conducted to further reveal the great potential of this receiver scheme. By using the verified noise model, we have addressed the sensitivity optimization issue of the receiver prototype. From the simulation results, we find non-monotonic evolution behavior of receiver sensitivity with respect to the parameter variation of the flux transformer. Based upon the optimal sensitivity, a considerable extension of the communication range is shown, in which a maximum communication range exceeding 100 m and a communication rate of several tens of kb/s could be simultaneously achieved in an underwater environment.

Overall, the results presented in this work have confirmed the significant advantages of deploying HTS-SQUID sensors in long-range MI applications due to its sensitivity advantages over other sensor technologies. This work shows great possibility for achieving a much-extended communication range by using the SQUID-based receiver. In addition, under the present portable scheme developed for practical applications, the total size of the receiver could be expected to be made smaller than a size of 0.1 m${}^{3}$, which could be suitable for many of the mobile application scenarios such as underwater or underground applications, or space-ground integrated information networks (SGIN), where both high sensitivity and high mobility are critically required. Lastly, the theoretically evaluated results presented in this work for achieving long-range MI communication and the prospects for underwater applications using the optimized SQUID receiver shall be experimentally demonstrated in our future works.

## Figures and Tables

**Table 1:** Characteristics of the constructed coils.

Coil Parameters	Pick-up Coil	Input Coil	Transmitter (Squared Coil)
Radius	25 mm	10 mm	500 mm
Coil turns	10	100	3
Wire diameters	0.57 mm	0.08 mm	-
Resistance	0.1 Ω	2.6 Ω (77 K)	5 Ω (300 K)
Inductance	9.8 μH	0.3 mH	15.7 μH

**Table 2:** Optimal pick-up coil turns and the corresponding field sensitivities at frequency of 10 kHz, 1 kHz, and 100 Hz for different coil radius $r_{p}$.

Receiver Parameters		$r_{p}=25$ mm	$r_{p}=100$ mm	$r_{p}=300$ mm
Optimum coil turns	10 kHz	80	30	15
	1 kHz	175	75	35
	100 Hz	500	290	150
Optimum field sensitivity (fT/$\sqrt{\mathrm{Hz}}$)	10 kHz	71.7	8.9	1.7
	1 kHz	140	16.5	3.3
	100 Hz	640	82	20

## Data Availability

The data that support the results of this study are available from the corresponding author, upon reasonable request.
